# Feasibility of Implementing Physical Activity Behavior Change Counseling in an Existing Cancer-Exercise Program

**DOI:** 10.3390/ijerph182312705

**Published:** 2021-12-02

**Authors:** Emma L. McGinnis, Laura Q. Rogers, Christine A. Fruhauf, Catherine M. Jankowski, Mary E. Crisafio, Heather J. Leach

**Affiliations:** 1 Department of Health and Exercise Science, Colorado State University, Fort Collins, CO 80523, USA; emma.mcginnis@colostate.edu (E.L.M.); mary.crisafio@colostate.edu (M.E.C.); 2 Division of Preventive Medicine, University of Alabama at Birmingham, Birmingham, AL 35205, USA; lqrogers@uabmc.edu; 3 Department of Human Development and Family Studies, Colorado State University, Fort Collins, CO 80523, USA; christine.fruhauf@colostate.edu; 4 College of Nursing, University of Colorado Anschutz Medical Campus, Aurora, CO 80045, USA; catherine.jankowski@cuanschutz.edu; 5 Department of Community and Behavioral Health, Colorado School of Public Health, Colorado State University, Fort Collins, CO 80523, USA

**Keywords:** cancer, exercise, implementation, pragmatic, behavior change, social cognitive theory

## Abstract

Purpose: This study examined the feasibility and acceptability of implementing research-tested physical activity (PA) behavior change counseling (BCC) sessions in an existing cancer-exercise program, and the preliminary effects on cancer survivor’s self-efficacy and PA. Methods: Participants were cancer survivors undergoing or within six-months of completing cancer treatment(s), and exercise program staff. Cancer survivors were randomized to receive the exercise program plus PABCC, or the standard exercise program. Feasibility and acceptability were assessed by recruitment, adherence, satisfaction, and a focus group with program staff. Qualitative data were analyzed using descriptive thematic analysis. Self-report questionnaires measured PA and exercise self-efficacy. Results: Recruitment was 33 out of 93 (36.7%), and *n* = 13 (39%) provided post-program data. Cancer survivors enjoyed PABCC sessions, but reported face-to-face delivery was an added time burden. Program staff expressed desire to implement PABCC, but perceived staff capacity and time as barriers to sustainability. Exercise self-efficacy increased by 21.5% in the PABCC group vs. 4.2% in the control. PA increased by 81.3% in the PABCC group vs. 16.6% in the control group. Conclusions: Implementing PABCC in an existing cancer-exercise program was acceptable and promising for increasing moderate to vigorous PA, but additional research is needed to enhance the feasibility and sustainability of translating efficacious behavioral interventions into existing cancer-exercise programs.

## 1. Introduction

Advancements in prevention and treatment have improved morbidity and mortality following a cancer diagnosis, resulting in a rapidly growing population of cancer survivors; defined as an individual living with cancer from diagnosis until end of life [1]. Many cancer survivors experience adverse diagnosis and treatment related side effects that negatively impact their physical and mental health, making it imperative to determine how to positively influence cancer survivor’s health-spans [1]. Fortunately, decades of research have demonstrated that exercise can improve physical and psychological outcomes relevant to cancer survivors including fatigue, physical function, and quality of life [2]. While breast cancer research has predominated the field of exercise oncology, literature is rapidly expanding to support such outcomes for a multitude of cancer types [2].

The robust evidence base supporting the benefits of exercise along the cancer continuum has led to numerous cancer-specific exercise programs offered in real-world, pragmatic settings such as community, rehabilitation, and clinical settings across the USA [3]. These programs have demonstrated success in improving health outcomes including physical function, fitness, and quality of life [3,4,5,6]. However, in order for cancer survivors to achieve the health benefits associated with exercise, adherence to exercise programs via increased levels of moderate to vigorous physical activity (PA) is necessary. Thus, to ensure adequate levels of PA are achieved during exercise programs, and possibly even after the program ends, theory and evidence-based PA behavior change techniques (BCT’s) should be utilized [7,8].

Several randomized controlled trials in cancer survivors have demonstrated efficacy for increasing PA [9] and PA maintenance [7], but few existing community or clinical exercise programs for cancer survivors have examined PA as an outcome [6]. Thus, the effectiveness of pragmatic cancer-exercise programs to enhance PA adoption or maintenance remains in question and may be related to a lack of translation of hypothesis-driven, theory-based PA behavior change interventions to real-world settings (e.g., clinic or community).

To date, several studies have concluded that interventions utilizing a theoretical framework, such as the social cognitive theory [10], and behavior change techniques (BCT’s) such as goal setting, instruction on how to perform behavior, setting of graded tasks, social support, and self-monitoring, are effective for PA adoption and maintenance in cancer survivors [7,9]. Unfortunately, there is a lack of translation of theory-based PA interventions and the use of BCT’s to exercise programs for cancer survivors that are delivered outside of highly controlled research environments. For example, a 2019 review of community-based cancer-specific exercise programs reported that although 42% of the programs included at least one education and/or discussion component, less than 20% utilized a behavior change theory to develop or deliver the program [6]. The reason for this may be that often, PA interventions and randomized controlled trials designed to test efficacy are time and resource intensive, and do not take into account contextual factors of real-world settings such as personnel and cost. This results in limited transferability or “dissemination” of PA behavior change interventions conducted in research or laboratory-based settings to exercise programs delivered in the clinic or community. Currently, it is unknown what the specific challenges are for existing cancer-specific exercise programs to adopt and implement theory-based BCT’s to help their participants increase and maintain PA. Therefore, the purpose of this study was to examine (1) the feasibility and acceptability of implementing evidence-based PA behavior change counseling (PABCC) in an existing exercise program for cancer survivors, and (2) changes in self-efficacy and PA from pre-to post-program.

## 2. Materials and Methods

### 2.1. Study Design

This pragmatic randomized controlled trial (NCT03976193) recruited cancer survivors who were enrolled in an existing exercise program. During the program’s baseline assessment, survivors were presented with the option to participate in the study, where they would be randomly allocated by the study coordinator to receive either the current exercise program (control), or the current exercise program plus six PABCC sessions (intervention). Randomization was 1:1, using a research randomization website, in blocks based on the program’s monthly enrollment (i.e., at the end of each month, enrolled participants were randomized). Study staff and participants were not blinded to group allocation. If individuals declined to participate in the study, they received the current exercise program. Inclusion criteria were matched to the existing program: >18 years old, diagnosed with cancer, currently receiving or within six-months of receiving active cancer treatment (chemotherapy, radiation therapy, or surgery) at the University of Colorado Cancer Center, and have a signed physician’s clearance to participate in a supervised exercise program. The only additional study eligibility criteria were the ability to attend a minimum of five out of six PABCC sessions held at the same health and wellness facility as the exercise program. Participants were enrolled from July 2019 to February 2020 and signed an informed consent document.

Additionally, exercise program staff were asked to participate in an audio-recorded focus group during the final month of the study. Exercise program staff included those involved in the day-to-day operations of the exercise program and were each credentialed exercise professionals with a Bachelor of Science in exercise science or kinesiology. Exercise program staff signed an informed consent document prior to participating in the focus group. All study procedures were approved by the University’s review board for the protection of human subjects (COMIRB #19–0323).

### 2.2. The Exercise Program (Control)

BfitBwell has been implementing exercise training for cancer survivors at the Anschutz Health and Wellness Center since 2013 (https://medschool.cuanschutz.edu/colorado-cancer-center/bfitbwell, accessed on 27 November 2021), and has demonstrated effectiveness for improving physical fitness, fatigue, and depression [11]. Program details have been published previously [11]; therefore, only pertinent information about the program is described herein.

BfitBwell is a three-month exercise program, which includes individual pre- and post-program assessments, twice weekly one-on-one exercise sessions (Month 1), and small group exercise sessions (up to four participants, Months 2 and 3) led by a certified exercise physiologist/cancer exercise specialist and/or supervised intern. Each exercise session is approximately 50 min in length, and focuses on a combination of resistance, aerobic, and flexibility exercises individualized to each participants’ fitness and medical needs, and exercise preference. Exercise intensity is prescribed based on an individual’s health and symptom status and is monitored using a rating of perceived exertion (RPE) scale of 0–10 [11]. The exercise instructor adjusts exercise intensity per subjective reporting to maintain an RPE of less than 8 and adapts exercises to accommodate fluctuations in health due to acute disease or treatment-related symptoms [11]. The program is open Monday–Friday from 8 a.m. to 4 p.m., and costs USD 59 per month. These costs were not subsidized for participants enrolled in the current study.

### 2.3. The Exercise Program and Physical Activity Behavior Change Counseling (PABCC) Sessions (Intervention)

Individuals randomized to the intervention group received the same exercise program as the control group (as described above), plus six PABCC sessions. PABCC sessions were adapted from a randomized controlled trial that demonstrated efficacy for increasing and maintaining PA among breast cancer survivors [12,13]. The intervention group received the same supervised exercise sessions as the control group, and individual and group-based PABCC sessions based on Social Cognitive Theory [14]. The PABCC sessions operationalized BCTs such as goal setting, barrier identification, self-monitoring, behavioral modification, time management, cognitive reframing, relapse prevention, and role models [12,13]. For the current study, PABCC session BCT content was not changed. Sessions were held once per week, every other week, for the duration of the 3-month BfitBwell program, in person, and at the same health and wellness facility that the BfitBwell program is held. Participants attended PABCC individually or in pairs based on their availability and the number of participants enrolled in the study at a given time. All PABCC sessions were facilitated by the first author and study coordinator (EM), who was trained on PABCC protocols by a former study staff member from the original randomized controlled efficacy trial. During the PABCC sessions, the study coordinator presented information using PowerPoint slides, facilitated discussion, and journaling prompts using a printed workbook. Other strategies included goal setting, and “role modelling” where a previous exercise program participant was invited to share their program experience with study participants.

### 2.4. Focus Group with Exercise Program Staff

A semi-structured focus group protocol was used to address the feasibility and acceptability of implementing PABCC sessions as part of the existing exercise program. Exercise program staff were selected for this focus group due to their expertise in delivering the BfitBwell exercise program, including knowledge of day-to-day functions, and program resources. The focus group lasted one hour and was conducted by the study coordinator and a research assistant who were both graduate students trained in qualitative research methods. The study coordinator spent the first 10 min of the focus group providing an overview of the PABCC session content to familiarize exercise program staff with the intervention. The study coordinator then facilitated the focus group by asking eight probing questions that were developed to address topics such as staff perception of PABCC implementation, feasibility to continue PABCC in the exercise program, and barriers or facilitators to PABCC continuation in the exercise program. The study coordinator led the focus group discussion while the research assistant audio recorded the session and took detailed notes on the responses from exercise program staff.

### 2.5. Measures

Feasibility and acceptability measures are detailed in Table 1. Briefly, these outcomes were assessed via the tracking of relevant intervention items by the study coordinator; a study evaluation questionnaire completed by cancer survivors who were randomized to, and had completed, the intervention; and a focus group with exercise program staff.

Self-efficacy was measured at pre- and post-program using the Barriers Specific Self-Efficacy Scale (BARSE) to determine perceived capability to exercise in the face of commonly identified barriers to participation, and the Exercise Self-Efficacy Scale (ESE) to determine perceived capability to exercise a set frequency and duration at different time points [15,16]. Both of these questionnaires have been tested for reliability and validity in a variety of populations including middle-aged and older adults [15,16].

PA was measured at pre-and post-program using an adapted version of the Godin Leisure Time Activity Questionnaire [13,17], a widely used self-report measure of PA that has been shown to be valid and reliable [18,19]. The adapted version of the Godin was used to mirror what was used in the original randomized controlled efficacy trial from which the PABCC sessions were adapted [12,13]. Participants reported the frequency (days per week) and duration (minutes per session) of light, moderate, and vigorous aerobic exercise in a typical week over the past month. Weekly minutes of moderate to vigorous PA (MVPA) were calculated using the following equation:

(Minutes of moderate PA × Days of moderate PA) + (Minutes of vigorous PA × 2) × Days of vigorous PA

### 2.6. Statistical Analyses

Descriptive statistics were reported via means and standard deviations (*M ± SD*) or frequencies (*n*, %) as appropriate. To evaluate sample representativeness, independent t-tests compared demographic characteristics between study participants, and individuals who participated in the exercise program from September 2016 to April 2020. Means, standard deviations, and average within group change from pre-to post-program were calculated for self-efficacy and PA. Hypothesis testing was not performed on PA or self-efficacy measures because the study was not adequately powered to detect statistical significance in differences between groups. A priori sample size was based on the primary aim of examining feasibility and acceptability of implementation of PABCC sessions in the existing program.

Qualitative data from the audio recorded focus group and open-ended responses from the cancer survivor study evaluation questionnaire were analyzed using descriptive thematic analysis [20,21]. The audio-recorded focus group was transcribed verbatim by the study coordinator following the focus group. The transcript content was then reviewed and coded using inductive reasoning independently by the study coordinator and research assistant prior to being compared. The study coordinator and research assistant then compared codes and reviewed for discrepancies, defined as sections of text coded inconsistently between parties, and were settled via discussion between both researchers. Five coding discrepancies occurred; consensus was reached on all discrepancies after discussion between researchers, and no peer review was required. Codes were then tallied based on repetition and when a group of codes were repeated in a patterned way delineating repetitive topics, they were categorized into themes or subthemes. Themes and subthemes were determined based on quantity of tallies. Quotations were extracted and synthesized corresponding to the themes and subthemes to exemplify ideas that emerged from the focus group.

Open-ended responses from the cancer survivor study evaluation questionnaire were transcribed and coded following the same descriptive thematic analysis as the program staff focus group. Responses were reviewed and coded openly and independently by the research coordinator and research assistant, then reviewed for discrepancies in coding. No discrepancies occurred. Codes were then categorized into themes and subthemes using inductive reasoning based on similarities or patterns within responses. Quotes corresponding to themes and subthemes were extracted to represent the data.

## 3. Results

**Figure 1:**
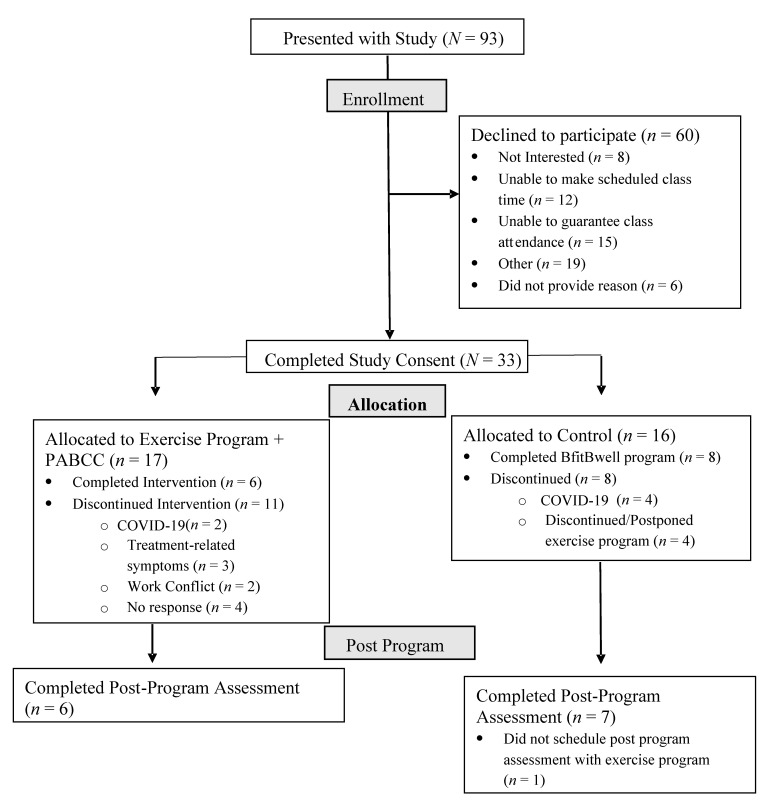
Consort Diagram. Flow of participants through study including reasons for declining participation and withdrawment.

A total of *N* = 93 exercise program participants were presented the study, and *n* = 33 (35.5%) consented to participate and were randomized. Reasons for not participating and withdrawal from the study are shown in Figure 1. Completion rate was 35.3% and 43.8% for the PABCC and control groups, respectively. Average attendance at PABCC sessions was 5.33 ± 0.52 (out of six).

Participant characteristics and representativeness are displayed in Table 2. There were no significant differences in sex, age, race, cancer diagnosis, treatment status, or body mass index (BMI) between study participants, and past exercise program participants.

Adaptations to PABCC sessions included changing workbook and presentation slides to remove and replace references to the randomized controlled trial with the current exercise program, and breast cancer specific language was changed to address all cancer types. Time and resources required for implementing the PABCC sessions included printing study workbooks (15 books @ USD 21.75 per book = USD 326.25), 12 h spent adapting sessions, 2 h training exercise program staff on informed consent procedures, 52 h preparing for PABCC sessions (e.g., randomization, participant email correspondence, and PABCC session preparation), and 39 h spent delivering PABCC sessions. Process fidelity was assessed using a check list of 28 items (Supplementary File S1), all items were completed “some of the time” or “most of the time”.

Pre- and post-program self-efficacy and PA values are displayed in Table 3. PABCC group participants reported a 21.5% increase (*M* = 10.0 ± 15.68) in barriers self-efficacy compared to a 4.2% increase (*M* = 2.38 ± 10.80) in the control group. PABCC group participants reported an 18.4% (*M* = −14.58 ± 30.31) decrease in exercise self-efficacy compared to a 4.3% decrease (*M* = −3.75 ± 17.15) in the control group. PABCC group participants reported an 81.3% increase in minutes per week of MVPA (M = 108.33 ± 166.5 min), compared to a 16.6% increase (*M* = 38.57 ± 114.6) in the control group. In the PABCC group, 67% (*n* = 4) reported an increase of ≥ 60 min per week of MVPA, compared to 25% (*n* = 2) participants in the control group.

### 3.1. Study Evaluation Questionnaire

The post-program study evaluation questionnaire was completed by (*n* = 6) PABCC group participants. Quantitative responses are shown in Table 4.

Descriptive thematic analysis of the open-ended questions revealed three themes and six subthemes:

**Theme** **1.** *Beneficial attributes of PABCC. Based on responses to: (1) what components of PABCC were beneficial; (2) the highlights of participating; and (3) any additional feedback, participants responded that social interaction, barrier identification, role models, and behavioral strategies addressing long term PA were benefits of participating in PABCC sessions.*

*“I really liked hearing from [role model]. Testimonials from old participants is inspiring.”*

[4]

*“I thought the sessions were very helpful and reinforced the importance of lifelong exercise…and its benefits on overall happiness.”*

[2]

*“Barriers-identifying and talk about possible solutions. Positive aspects-why exercise is good and helps me feel better.”*

[3]

*“Informal discussion with facilitator and other participant, inspiring visit from [role model], the Bfit alum.”*

[5]

**Theme** **2.** *Positive PABCC Facilitator feedback. When asked to provide additional feedback for the facilitator, participants provided positive responses regarding their experience in PABCC.*

*“You’re [facilitator] really good at bringing people back to topic in a nice and patient way.”*

[1]

*“I like all the interaction.”*

[3]

**Theme** **3.** *PABCC suggestions. Based on responses to questions asking (1) if they could change one aspect of PABCC to better suit their needs; (2) if any content should be covered in less detail; and (3) for additional feedback, survivors reported PABCC felt redundant at times.*

*“There was a lot of redundancy that made some of the sessions less appealing to me.”*

[4]

*“There were some aspects that seemed formulaic like questions/responses.”*

[2]

Participants also suggested offering a remote PABCC delivery modality to ameliorate the scheduling and location barrier.

*“Be closer to my home HA!”*

[1]

*“Have some of the sessions via Skype or Zoom to avoid having to drive to Anschutz.”*

[5]

*“Schedule it so that it could be done remotely so it wouldn’t complicate my schedule.”*

[3]

### 3.2. Focus Group with Exercise Program Staff Results

The focus group conducted with *n* = 4 exercise program staff members revealed four themes and thirteen subthemes regarding their perceptions on the feasibility and acceptability of implementing PABCC sessions as part of the standard program, their intent to continue, and perceived barriers to implementation. Themes and subthemes with representative quotes are presented in Table 5.

## 4. Discussion

This study examined the feasibility and acceptability of implementing evidence-based PABCC sessions in an existing exercise program for cancer survivors. Findings were mixed, with positive feedback about the session content at both the individual (i.e., participants) and setting (i.e., program staff) level; however, challenges remain in implementing PABCC sessions designed for a randomized controlled efficacy trial in a ‘real-world’ setting. This study also examined changes in self-efficacy and MVPA and found a larger magnitude of increase in barriers self-efficacy and weekly minutes of MVPA among those who were randomized to PABCC, no between-group conclusions were drawn due to the study sample size.

For those completing the exercise program and PABCC (intervention), acceptability of the PABCC sessions was supported by high adherence (89%), and positive responses on the study evaluation questionnaire. All participants enjoyed the PABCC sessions and indicated confidence in their ability to engage in independent PA following the sessions. Open-ended responses highlighted social support and barrier identification as positive attributes of PABCC sessions. The focus group with exercise program staff revealed they were supportive of implementing PABCC sessions in the program based on positive feedback they received from participants, and professional agreement towards the benefits of PABCC. Conversely, our findings raised questions surrounding the feasibility and sustainability of implementing PABCC sessions in the exercise program. Study recruitment rate (35%) was lower than anticipated. Given the minimal exclusion criteria beyond existing requirements for the exercise program, we expected a recruitment rate of ≥ 80%. Common reasons for declining participation in the study included “unable to guarantee attendance” and “unable to make class time”, and 83.3% of PABCC group participants indicated that attending PABCC sessions was an added time burden, and the location was inconvenient. The focus group with exercise program staff also revealed barriers to continuing to deliver PABCC sessions as part of the program, with the primary concern owing to staff capacity and time. Staff provided suggestions for alternative implementation strategies (e.g., video recordings of PABCC sessions, incorporating session content into face-to-face education sessions already being delivered). Taken together these findings indicate that the face-to-face delivery modality of PABCC sessions may not be feasible for cancer survivors already participating in face-to-face exercise sessions.

In terms of effectiveness, although the PABCC group showed a larger magnitude of improvement in barriers self-efficacy and MVPA from pre- to post-program than the control group, attrition and sample size prohibited a between-group comparison. Compared to the original randomized controlled efficacy trial from which the PABCC sessions were adapted [12], the magnitude of increases was much smaller in the current study. However, several factors, such as differences in treatment status, cancer type, baseline PA levels, and exercise session content, preclude direct comparisons in self-efficacy, barriers self-efficacy, and MVPA changes between the current study and the randomized controlled trial.

Previous studies in exercise oncology have identified similar implementation challenges including financial and administrative barriers based on an organization’s resources, and the need for further intervention adaptations to accommodate these barriers [20]. Findings from the current study suggest that despite positive feedback and support from cancer survivors and exercise program staff, further adaptations to PABCC sessions may be necessary for continued implementation in the current exercise program. Themes that emerged from the focus group with staff, such as a need for strong integration and collaboration between researcher, healthcare professional, and stakeholder for successful implementation, are also congruent with previous literature [22,23].

### Strengths and Limitations

This study was unique as it was the first to implement evidence-based PABCC sessions into an existing exercise program for cancer survivors. Strengths of this study included capturing data at both the individual and setting level, dissemination of an intervention in a real-world setting, use of a heterogeneous cancer population, and the use of the ‘standard of care’ exercise program as a comparison group. Another strength of this study was the use of a theoretical framework [24,25] to guide measures of feasibility, allowing information related to external validity such as reach and setting level data to be collected. While our small sample size and attrition rate provided feasibility information, it limited our ability to conduct between-group statistical analysis and draw conclusions about effectiveness. Another limitation included the emergence of the COVID-19 pandemic during the study, which impacted the study completion rate.

## 5. Conclusions

Based on the current literature which supports the efficacy of theory-based BCT’s to increase PA adoption and maintenance in cancer survivors [7,9], existing real-world exercise programs should consider incorporating BCT’s to support long-term, habitual PA in cancer survivors. This study aimed to address the gap in translating evidence-based PABCC sessions from a randomized controlled efficacy trial to a real-world exercise program for cancer survivors. Although this study presented a step forward in closing this research to practice gap, more studies are needed to determine how to adapt and implement PABCC sessions or incorporate BCT’s into existing cancer-exercise programs. Such future studies should adopt pragmatic designs such as hybrid implementation–effectiveness, and use established dissemination and implementation frameworks such as Reach, Effectiveness-Adoption, Implementation, Maintenance (RE-AIM) [24,26] or the Consolidated Framework for Implementation Research [27,28].

In conclusion, findings from this study highlight the challenges in implementing research-tested intervention strategies in real-world settings, and support the need for collaboration between researchers and practitioners across the translational research spectrum. Planning for and addressing contextual factors relevant to existing exercise programs when conducting theory-driven behavioral interventions may help narrow the research to practice gap in exercise oncology in order to promote long-term PA adherence among cancer survivors [23].

## Figures and Tables

**Table 1:** Measures of Feasibility and Acceptability.

	Who	What	How
Acceptability	Participants	Factors influencing study participation	Number who enrolled in study out of number offered, and reasons for declining to participate
Acceptability	Participants	Adherence to PABCC sessions	Attendance tracking
Acceptability	Participants	Perceptions of delivery, facilitator, content, time burden, etc.	Study evaluation questionnaire including closed (i.e., Likert scale) and open-ended questions completed post-program
Feasibility	Participants	Representativeness	Compare study participant characteristics (i.e., sex, age, diagnosis, current treatment status, etc.) to previous participants enrolled in BfitBwell
Feasibility	Study Coordinator	Adaptations to PABCC sessions, time, costs	Tracking any changes to slides and handouts; hours training on study protocol and delivering PABCC sessions; costs
Feasibility	Study Coordinator	Process Fidelity (i.e., were the PABCC sessions delivered as planned)	Fidelity checklist completed after every PABCC session
Feasibility and Acceptability	Exercise Program Staff	Session content, delivery modality, staff training/time, appropriateness of perceived fit with current program, barriers to implementation, intent to continue	Focus Group

Abbreviations: PABCC, physical activity behavior change counseling.

**Table 2:** Representativeness of Study Participants.

	Study Participants *N* = 33 ^a^	Exercise Program Registry *N* = 524 ^a^
	*n* (%)	
Sex		
Female	21 (63.6%)	308 (63.6%)
Male	12 (36.4%)	176 (36.4%)
Race		
Asian	1 (3.1%)	19 (4.3%)
Black/African American	1 (3.1%)	21 (4.7%)
White	30 (93.8%)	373 (84.2%)
Cancer Diagnosis		
Breast	11 (39.3%)	141 (30.6%)
Hematological	5 (17.9%)	38 (8.2%)
Ovarian	2 (7.1%)	15 (3.3%)
Prostate	2 (7.1%)	37 (8%)
Other	8 (28.6%)	230 (49.9%)
On Treatment during Program		
Yes	21 (63.6%)	316 (64.2%)
No	12 (36.4%)	175 (35.6%)
	*Mean ± SD*	
Age (years)	54.3 ± 12.4	55.5 ± 14.1
Body Mass Index (kg/m^2^)	28.2 ± 7.2	26.9 ± 6.2

^a^ n’s do not add up to 33 or 524 for all measures due to missing data.

**Table 3:** Self-efficacy and Physical Activity.

	Exercise Program + PABCC (*n* = 6)		Control (*n* = 7)	
Measure	Pre	Post	Pre	Post
Exercise Self-Efficacy (ESE) ^a^	79.2 (27.4)	64.6 (28.7)	88.2 (12.6)	88.5 (14.7)
Barriers Self-Efficacy (BARSE) ^b^	46.5 (20.4)	56.5 (18.6)	56.2 (25.8)	58.6 (26.6)
MVPA (minutes per week)	133.3 (48.0)	241.7 (160.3)	232.9 (317.9)	271.4 (321.1)

Abbreviations: MVPA, moderate to vigorous physical activity. All data are displayed as mean (standard deviation). ^a.^ Exercise Self-Efficacy (ESE) scale: 0–100 with a higher score indicating better self-efficacy. ^b.^ Barriers Self-Efficacy (BARSE) scale: 0–100 with a higher score indicating better self-efficacy.

**Table 4:** Study Evaluation Questionnaire (*n* = 6).

Question	Answer: Probably Yes, Yes, or Definitely Yes *n* (%)
Did you enjoy the behavior change counseling sessions?	6 (100%)
Was attending the behavior change counseling sessions an added time burden to you?	5 (83.3%)
Do you think attending behavior change counseling sessions improved your ability to continue exercising after the end of the BfitBwell program?	5 (83.3%)
Did the facilitator and group environment of the behavior change counseling sessions provide you with a sense of community and support that you found beneficial?	6 (100%)
Did the facilitator effectively deliver information and generate open discussion?	6 (100%)
After completing discussion sessions, do you feel confident that you have the knowledge and skills to exercise safely and effectively without professional guidance in another setting (e.g., home, fitness center, etc.)?	6 (100%)

**Table 5:** Themes, Subthemes, and Representative Quotes from Focus Group with Exercise Program Staff.

Themes and Subthemes	Question(s)	Representative Quotes
Theme 1: Positive Cancer Survivor Feedback		
Staff believe PABCC is beneficial to program and participants	(1) Please describe your thoughts about the integration of PABCC (2) Would it be possible for BfitBwell to continue delivering PABCC sessions as part of the program?	*“Everyone loves it and when I present it to them in the assessment with the consent, everyone’s like it’s a need and they’re very excited about it.”* [2] *“In fact, they [participants] almost don’t want to be randomized to the [control] group.”* [1] *“I think if we had the resources, we would do this no matter what the data says. We would start right now,”* [3] *“yeah absolutely”—unanimous agreement.* *“I mean selfishly I wish this were a standard of care for us already.”* [1]
PABCC sessions align with direction and mission of the facility	(1) Based on your experiences and knowledge of PABCC, how is this beneficial to the BfitBwell program?	*“The whole push for the center as a whole, is to incorporate into every program component of physical activity, mindfulness and nutrition. So, it kind of aligns with where the center is going.”* [1] *“It’s… a lot like [other program offered at facility], just in a different [population], working with cancer patients instead of weight loss…they have a behavior change component.”* [2]
Theme 2: Barriers to Implementing PABCC Sessions in the BfitBwell Program		
Staff Capacity	(1) What barriers or other factors would prevent BfitBwell from continuing PABCC as part of the program? (Time, staff, cost, equipment, resources, etc.)	*“Yeah, like if you [study coordinator] could come down and always teach it, it would be great because you have the flexibility, but that flexibility piece would totally go away if we were doing [it]… we are flexible with their workouts and… we run out of time there.”* [2] *“I think the short answer is Yes…”* [3] *[PABCC could be implemented], “long answer is how we allocate time”.* [4]
Exercise program interns not suitable for delivering PABCC sessions	(1) What barriers or other factors would prevent BfitBwell from continuing PABCC as part of the program? (Time, staff, cost, equipment, resources, etc.) (2) If BfitBwell were to continue using PABCC, who would deliver these sessions to cancer survivors?	*“The biggest factor with [interns delivering PABCC] is that so many of them are coming in with absolutely no knowledge of cancer, and that’s ok. But to then have someone like really green like that administer a class on ya know time management, and barriers to exercise, and then answering all of the cancer specific [questions]. I think that would be a big undertaking for a green individual in the industry.”* [1] *“I don’t know that an intern could do it.”* [3]
Cost to hire new staff	(1) What barriers or other factors would prevent BfitBwell from continuing PABCC as part of the program (e.g., time, staff, cost, equipment, resources, etc.)?	*“and the cost of if we had to hire a staff… then we couldn’t afford that.”* [2]
Contribution of additional resources from Cancer Center or Wellness Center	(1) What would motivate the Cancer Center to invest additional resources into implementing PABCC into the standard BfitBwell program?	*“I mean it is [program champion’s] biggest push for us is to focus on the research rather than the number of patients that pass through, so I know that long term they care about the data we are collecting and that it’s quality data.”* [1]
Accessibility to survivors	(1) What barriers or other factors would prevent BfitBwell from continuing PABCC as part of the program? (Time, staff, cost, equipment, resources, etc.)	*“In fact, that’s been…the biggest barrier to someone not joining [the study] is the conflict of the dates or times when the sessions are offered.”* [1] *“I think again it’s like the timing, cause some people are like I can only come at 8 because I work, I can only come at 3 because I work, I can only come at lunch cause of this.”* [3]
Theme 3: Alternative PABCC Session Implementation Strategies		
Alternative delivery modality	(1) What could be done to reduce the cost of implementing PABCC as part of the standard BfitBwell program? (2) Would it be possible for BfitBwell to continue delivering PABCC as part of the program?	*“Like a version 2.0 of our classroom session…maybe it’s a 3-part series and they come to a, b, and c.”* [1] *Video option* *“Like you’re recording a lecture or a live, yeah.”* [2] *“Here’s a link to a YouTube and keep it private with that same link.”* [4]
Current staff optimal delivery personnel	(1) If BfitBwell were to continue using PABCC, who would deliver these sessions to cancer survivors? (2) Would it be possible for BfitBwell to continue delivering PABCC as part of the program?	*“I think it would have to be…one of the four of us…I don’t know how we could make it work.”* [2] *“and the cost of if we had to hire a staff… then we couldn’t afford that.”* [2]
Fee for service	(1) What could be done to reduce the cost of implementing PABCC as part of the standard BfitBwell program? (2) Would it be possible for BfitBwell to continue delivering PABCC as part of the program?	*“The only other thing that I would be interested in doing like a fee for service even if was nominal. Like if we roll it into their membership costs so it doesn’t feel like they are paying for something extra, but just a way for us to pull a little bit of revenue so that we could support an additional staff person.”* [1] *“That [fee for service] would get solid members too, if they pay, they are more likely to come to this. We are not wasting the hour and only one person shows up.”* [2]
Hire intern in alternative field	(1) What could be done to reduce the cost of implementing PABCC as part of the standard BfitBwell program? (2) If BfitBwell were to continue using PABCC, who would deliver these sessions to cancer survivors?	*“The idea of an intern that saves us a lot of costs like we’ve had interns that come to us that are…psychology majors…that could be something we like broaden our intern take from…we’ve turned them away mostly because they don’t fit any of our criteria but if that was their project, their whole internship is like developing this, working on this class, offering it more.”* [2] *“Or like a mph student…might take this on for like a master’s program.”* [3]
Theme 4: Collaboration between Healthcare Professionals		
Lack of perceived value of exercise by physicians	(1) What barriers or other factors would prevent BfitBwell from continuing PABCC as part of the program? (2) What would motivate the Cancer Center to invest additional resources into implementing PABCC into the standard BfitBwell program?	*“But from our experience, do providers all believe this? No. Like the referees need to get on board.”* [1] *“I don’t feel like everyone is even sold on the fact that cancer patients have to exercise long term, or anything like that, so it’s like a two part, yes exercise improves quality of life for your cancer patients, and this is why they need to continue long term.”* [2] *“We get more referrals from mid-levels, nurse schedulers, ‘PA’s* [4]*’, PA’s and self-referrals than we do from [oncologists]. And if they don’t hear about it from their oncologist, then what we do hear is why didn’t my doctor tell me about this.”* [1]
Support for PABCC from program advocates and Wellness Center leadership	(1) What would motivate the Cancer Center to invest additional resources into implementing PABCC into the standard BfitBwell program? (2) Is there anything else you would like to share about the BfitBwell program or PABCC?	*“Yeah, or an advocate like [program champion]. That’s why this program exists. There’s someone at the top who is like do this and waving that flag.”* [3] *“I also think someone like [cancer researcher]. Obviously, she’s seeing patients for what they are going through trauma wise with their diagnosis and everything like that…this kind of like a new avenue…but someone like her could be a really big champion for us if [current program champion] couldn’t necessarily be, I mean he would be all in, but she could potentially give more resource to that I think.”* [2]

## Data Availability

The data presented in this study are available on request from the corresponding author. The data are not publicly available due to policies set forth by the institutional review board for the protection of human subjects.

## Supplementary Materials

The following are available online at https://www.mdpi.com/article/10.3390/ijerph182312705/s1, File S1: Fidelity Checklist; File S2 Consort 2010 Checklist of Information to Include When Reporting a Pilot or Feasibility Trial. Reference [29] is cited in the supplementary materials.
